# Prevalence of the Patterns of Unhealthy Diet in the School and University Students of Iran: A Systematic Review and Meta-Analysis

**DOI:** 10.1155/2024/2697001

**Published:** 2024-07-30

**Authors:** Seyyed Amir Yasin Ahmadi, Neda SoleimanvandiAzar, Mahshid Roohravan Benis, Ali Mehrabi, Roya Vesal Azad, Marzieh Nojomi

**Affiliations:** ^1^ Preventive Medicine and Public Health Research Center Psychosocial Health Research Institute Department of Community and Family Medicine School of Medicine Iran University of Medical Sciences, Tehran, Iran; ^2^ School of Public Health Iran University of Medical Sciences, Tehran, Iran

## Abstract

**Introduction:**

The present study was conducted to investigate the pooled prevalence rate of the different patterns of unhealthy diet among the school and university students of Iran.

**Methods:**

In this systematic review, the type of the main question was regarding prevalence and the effect measure was prevalence rate reported along with 95% confidence interval (CI). Data bases including PubMed, Scopus, and Web of Science as well as Google Scholar and Persian resources were used. The Newcastle–Ottawa scale (NOS) checklist was used for quality assessment of studies.

**Results:**

The extracted types of unhealthy diet in the present systematic review were “breakfast skipper,” “fast food,” “hydrogenated oils consumption,” “salty snacks,” “sweetened beverages,” “breakfast skipper,” “dinner skipper,” “launch skipper,” and “sweets.” The range of pooled prevalence for different types was 0.06–0.75. The data of 16,321 subjects included in six studies were analyzed. The pooled prevalence of unhealthy diet was 0.28 (95% CI: 0.23–0.33, *I*^2^ > 99%) overall, 0.25 (95% CI: 0.20–0.31, *I*^2^ > 99%) in school students and 0.37 (95% CI: 0.12–0.62, *I*^2^ > 99%) in university students. The most prevalent pattern was breakfast skipping 0.39 (95% CI: 0.28–0.50) followed by consumption of sweetened beverages 0.31 (95% CI: 0.20–0.43). The pooled prevalence range among the patterns was 0.06–0.75 (random effects for all).

**Conclusion:**

The pooled prevalence was 28% for unhealthy diet among the Iranian students (6% to 75% in different patterns). Although there was uncertainty regarding the pooled evidence, the whole of the mentioned range was clinically important for health policymakers. Decisions should be made on the basis of the patterns.

## 1. Introduction

Adolescence is a period that has a high prevalence of risky behaviors such as drug and alcohol use, inadequate sleep and lifestyle problems [1], and many unsuitable nutritional habits are formed during this period [2]. Many of noncommunicable diseases that occur in middle and old age are the result of unhealthy lifestyle and improper nutritional habits in childhood and adolescence [3].

The World Health Organization (WHO) states that proper nutrition is a cornerstone of good health [4]. Proper nutrition is a determining factor in health and ensuring adequate growth. Teenagers need to follow a well-designed diet due to their active lifestyle. Skipping meals, using salty and fatty foods, and consuming sweetened beverages are among the unhealthy eating habits among adolescents and school-aged children. Unhealthy eating habits at this age, when growth is fast and nutritional needs are greater, can interfere with the growth and health of adolescents [5]. In a study that examined the habit of eating breakfast in school-aged children, it was seen that only 30.9% of participants did not intake full breakfast (six times or less) and 69.1% consumed a complete one [3]. In another study, the frequency of breakfast, lunch, and dinner consumption was 37%, 56.5%, and 46.1% in six days of a week, respectively. About 7.8% of these students used carbonated sweet drinks, 13.6% of salty snacks, and 34.1% of sweets on a daily basis [6]. Other studies have also been conducted in the field of breakfast consumption in primary school children, which have similar results and show that 8 [7], 8 [8], and 2.5 percent of these children [9] do not eat breakfast.

The importance of dietary patterns is not limited to the nutritional health. Many mental illnesses in adolescence are also related to the dietary pattern of adolescents [10]. It is believed that nutritional status in children and adolescents is associated with psychiatric disorders as food intake has an effect on brain development and function. For instance, neurodevelopmental disorders may be associated with prenatal and childhood intake of essential fatty acids [11]. A school-based cross-sectional study in a large population of Brazilian adolescents showed that different patterns of unhealthy diet are associated with common mental disorders [12]. On the basis of the present evidence, it is expected that unhealthy diet in childhood, adolescence, and youth is a health issue. According to the role of social and cultural status in dietary patterns [13], the mentioned issue may become a specific problem in some countries and populations. Knowing the frequency and patterns of unhealthy diets is an important step to resolve the stated problem. Unhealthy diet may have different patterns and examples, and each of them has its own prevalence. Such prevalence rates can be reported as range or pooled rates. Although the patterns are different, it is not without grace to pool the results.

As stated, unhealthy diet in adolescents is a major problem in the world and is categorized as a high-risk behavior. Iran as a developing country is not an exception. So far, there are some large cohort studies such as “Childhood and Adolescence Surveillance and PreventIon of Adult Non-communicable disease” (CASPIAN) conducted in Iran in this regard [14, 15]. However, a meta-analytic approach was needed to pool the results of different studies. The present study was conducted to investigate the pooled prevalence rate of the different types of unhealthy diet among the students of Iran.

## 2. Materials and Methods

### 2.1. Study Design

The present systematic review and meta-analysis was conducted according to Preferred Reporting Items for Systematic Reviews and Meta-Analyses (PRISMA) statement. The type of the main question was regarding prevalence, and the effect measure was the prevalence rate. This question consisted of the prevalence of different types of unhealthy diets in the context of students in Iran. The effect measure method was prevalence rate. The protocol of the study was approved by the Research Council of the Psychosocial Health Research Institute, Iran University of Medical Sciences, with registration number 13919.

### 2.2. Sources and Search Strategy

Our general search strategy was the following which was searched without limiting the search to the titles, abstracts, or keywords. Databases such as Scopus, PubMed, and Web of Science (WOS) were used. Since it was expected that there would be some important papers in Persian sources, Persian databases including Magiran, Scientific Information Database (SID), and Noor as well as Google Scholar were used by two independent researchers. The limitation of Persian sources was that the exact syntaxes were not properly worked, and therefore, the researchers searched manually and screened the first 50 results of each database. There was no time limitation. The search process was done in December 2022.

Search strategy: (Student OR Pupil OR college OR school) AND (Prevalence OR Epidemiology OR Incidence OR Frequency OR Outbreak OR Occurrence OR “Population-Based”) AND (Violence OR “Assaultive Behavior” OR “high risk behavior” OR “high risk behaviors”) AND (Diet^*∗*^ OR Feeding OR Eating OR Nutrition^*∗*^ OR Food^*∗*^) AND (Iran).

After removal of duplicates, the remaining documents were subjected to screening based on the titles and the abstracts by two independent researchers (MRB and AM) and investigation of a third researcher (SAYA) if necessary. After screening and adding the papers from Persian sources, citation search, and manual search in Google Scholar (by SAYA), the full texts were scrutinized for investigation of the eligibility criteria. The eligibility criteria included cross-sectional studies reporting prevalence rate of unhealthy diet (in general and *per* type), school or university student samples, and conducting the research in Iran. The age range was not regarded, but the participants should be school or university students. No time range was considered. These criteria were also subjected to data extraction. We extracted the patterns of unhealthy diet, the prevalence rates of these patterns, and demographic variables from the included studies.

### 2.3. Quality Assessment

Quality assessment of the studies was performed by the Newcastle–Ottawa scale (NOS) checklist for cross-sectional studies [16]. This checklist consisted of the parts' selection (in turn, including representativeness of the sample 0-1 point, sample size 0-1 point, nonrespondents 0-1 point, and ascertainment of the exposure 0–2 points), comparability 0 and 2 points, and outcome (in turn, including assessment of outcome 0–2 points and statistical tests 0-1 point). The total scoring categories were unsatisfactory (<5 points), satisfactory (5-6 points), good (7-8 points), and very good (8–10 points). The process was conducted by two independent researchers.

### 2.4. Statistical Analysis

For meta-analysis, Stata 17 (Stata Corp. LLC, TX, US) was used with the latest version of packages *metan*, *metafunnel*, and *metareg*. Pooled prevalence rates were calculated for unhealthy diet divided by the pattern with their asymptotic 95% confidence intervals (CI). *I*^2^ test was used to investigate heterogeneity and random effects model was used if *I*^2^ > 50% with *P* < 0.05. Subgroup analysis was performed with *-by()-*option of the command. The results were reported as forest plots. Funnel plots were used for visual investigation of publication bias based on asymmetry of the distribution of the effect sizes. Sensitivity analysis was performed to investigate the effect of each study removal on the pooled prevalence. Cumulative meta-analysis and meta-regression were used to investigate the effects of the demographic variables and their trends on the pooled effect.

## 3. Results

### 3.1. Baseline Findings

**Figure 1:**
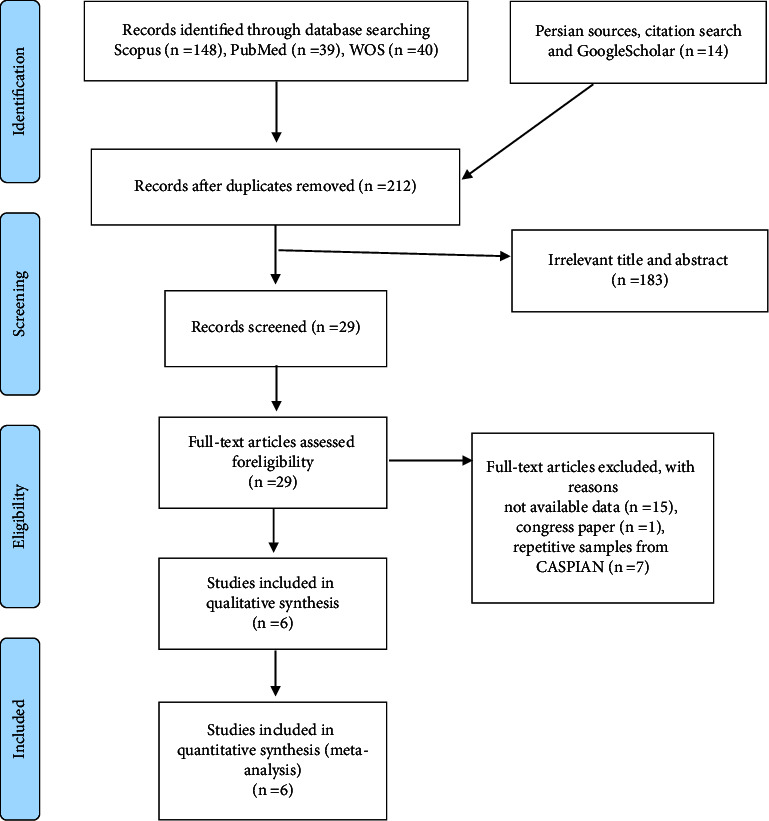
PRISMA flowchart.

According to the PRISMA flowchart (Figure 1), a total of six papers were eligible for meta-analysis [3, 6, 14, 15, 17, 18]. The time range of publication was from 2011 to 2018. One study investigated the outcome among the university students, while the other studies were among school students. The smallest sample size was 289, while the largest sample size was 13486 that was for a famous cohort called CASPIAN (totally, the data of 16,321 cases were analyzed). The extracted patterns were classified as “breakfast skipper,” “dinner skipper,” “fast food,” “hydrogenated oil consumption,” “lunch skipper,” “poor nutrition,” “salty snacks,” “sweetened beverages,” and “sweats.” The most common pattern was “breakfast skipper” as there were five studies for this pattern, followed by “sweetened beverages” and “salty snacks” with four studies for each. According to the quality assessment, all the studies were at least satisfactory. The baseline information of the studies is shown in Table 1.

### 3.2. Pooled Analysis

**Figure 2:**
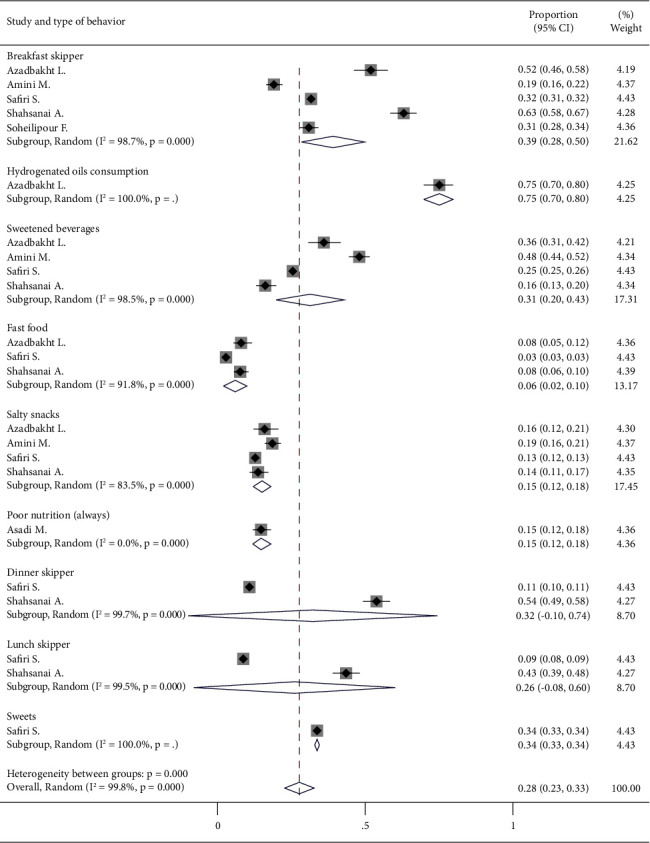
Forest plot for prevalence of unhealthy diet with subgroup analysis based on the types of unhealthy diet.

**Figure 3:**
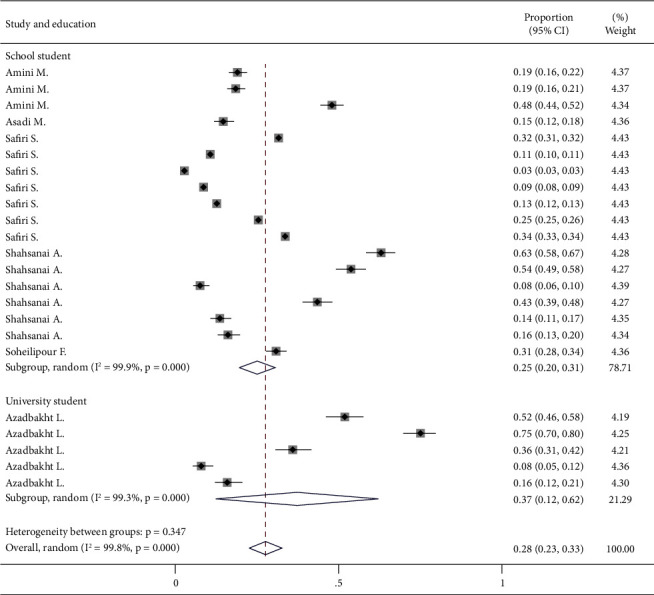
Forest plot for prevalence of unhealthy diet with subgroup analysis based on education.

A total of nine study types of unhealthy diet groups from six studies were analyzed (including 23 study–subgroups). A significant heterogeneity was found between the study type of unhealthy diet group (*I*^2^ > 99%, *P* < 0.001). Because of heterogeneity, large variation in sample sizes, and overlap of samples in the subgroups, a random effects model was considered. Accordingly, the pooled prevalence among all the study types of unhealthy diet groups was 0.28 (95% CI: 0.23–0.33). Pooled analysis was conducted *per* the type of unhealthy diet. Accordingly, among the subgroup analyses with three or more populations, fast food consumption showed the lowest prevalence 0.06 (95% CI: 0.02–0.10) followed by salty snacks 0.15 (95% CI: 0.12–0.18). The most prevalent pattern was breakfast skipping 0.39 (95% CI: 0.28–0.50) followed by consumption of sweetened beverages 0.31 (95% CI: 0.20–0.43). Other subgroups had fewer populations. The heterogeneity between the groups was statistically significant (*P* < 0.001) (Figure 2). In addition, subgroup analysis was performed for education. No significant difference was observed between university and school students (*P*=0.347) (Figure 3).

### 3.3. Publication Bias

**Figure 4:**
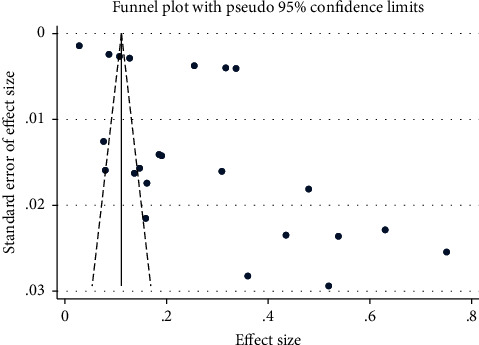
Funnel plot of publication bias assessment.

Funnel plots showed asymmetry for the distribution of the effect sizes of the smaller studies. Most of the studies were outside the funnel (Figure 4).

### 3.4. Sensitivity Analysis

**Figure 5:**
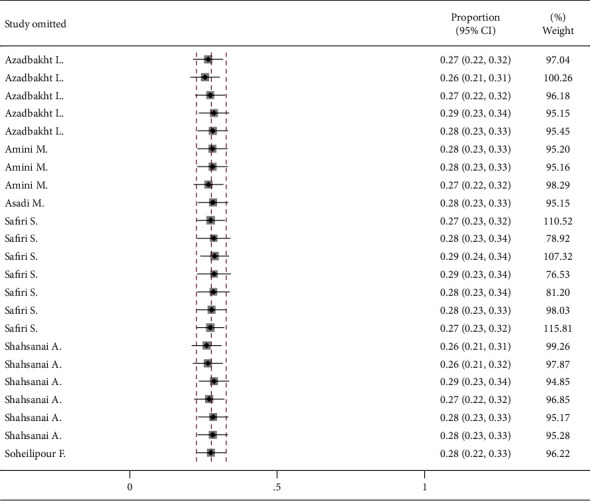
Sensitivity analysis for effect of each study type of unhealthy diet removal.

Pooled effect is shown for removal of each study type of unhealthy diet (leave-one-out approach). The lowest and highest possible effect sizes were 0.26 and 0.29, respectively (Figure 5). Subgroup analyses can also be interpreted as sensitivity analysis.

### 3.5. Cumulative Analysis and Meta-Regression

**Figure 6:**
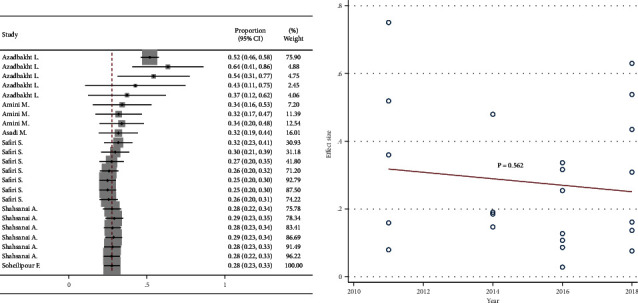
Cumulative meta-analysis and meta-regression for the trend of publication year effect.

**Figure 7:**
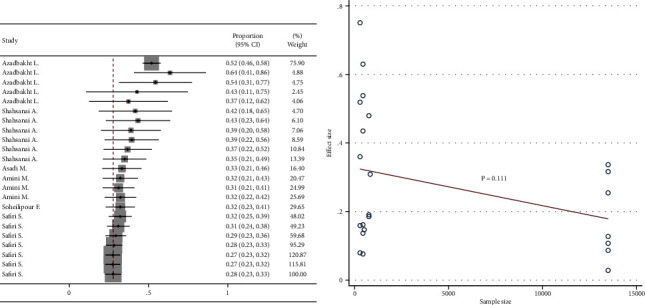
Cumulative meta-analysis and meta-regression for the trend of sample size effect.

**Figure 8:**
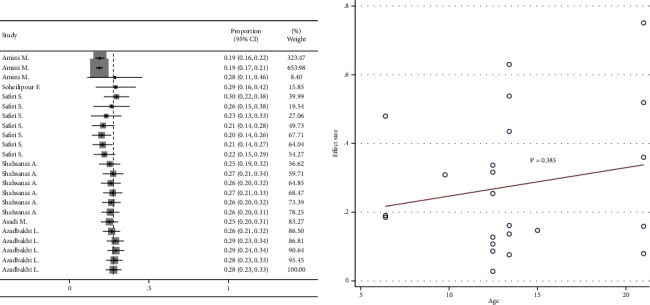
Cumulative meta-analysis and meta-regression for the trend of age effect.

**Figure 9:**
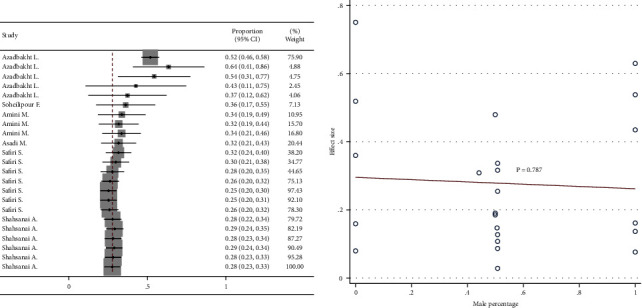
Cumulative meta-analysis and meta-regression for the trend of male proportion effect.

Cumulative meta-analysis showed a negative visual trend for the time of publication. However, this trend was not statistically significant based on meta-regression (Figure 6). Cumulative meta-analysis showed a negative visual trend for sample size. However, this trend was not statistically significant based on meta-regression (Figure 7). No visual pattern of trend was observed for age (Figure 8). Cumulative meta-analysis showed a negative visual trend for male proportion. However, this trend was not statistically significant based on meta-regression (Figure 9).

## 4. Discussion

According to the stated problem and the rationale, the present study was conducted to achieve the patterns of unhealthy diet in the literature, as well as the range and the pooled prevalence of different types of unhealthy diet in the context of Iranian students. A total of 16321 persons from six studies were investigated. Eight studies were found from the CASPIAN cohort, and we selected only one of which was the most complete and most recent. Studying the characteristics of overall diet resulted in the examination of the combined nutrient effects on health and better prediction of diet-disease relationships [19]. To our knowledge, this is the first systematic review and meta-analysis study to obtain prevalence of the different types of unhealthy diet among the students of Iran. The existing studies in Iran are mainly conducted on adults [20, 21].

In terms of risk of bias, all the included studies had at least a satisfactory quality. The comparability section of the checklist had a two-point item to adjust for confounders. Since the question of our study was prevalence, this item was not relevant, and as a result, none of our studies received these two points. Instead, studies that had subgroups based on the type of unhealthy diet were given one point. However, all the studies gave a satisfactory score or higher in spite of the mentioned limitation. In terms of heterogeneity, the source of heterogeneity could be attributable to the differences in subgroups (types of unhealthy diet). In terms of publication bias investigation on the basis of funnel plots, smaller studies showed a higher prevalence. It seemed that the gap of small studies with low prevalence indicated publication bias. However, it did not seem to have much effect on the pooled result.

In the worst scenario of the sensitivity analysis, by removing one study, the total frequency decreases from 0.28 to 0.26. The results of this part were also in line with the results of the funnel plot and indicated the possibility of a slight drop in the pooled frequency in the absence of publication bias. None of the baseline factors had a significant effect on the pooled results. Although age is logically subjected as an influencing factor, it could not affect the pooled result (*P*=0.385). Among these factors, the most effective one was the sample size, which showed a negative trend on the prevalence in the cumulative meta-analysis and funnel plot; however, no significant relationship was observed in the meta-regression (*P*=0.111).

Different patterns of unhealthy diet among the studies may be a potential source of heterogeneity. Comparing diet patterns between studies was somewhat difficult due to differences in dietary assessment, food groups, and the number of patterns that should be retained for analyses and statistical techniques. Despite inconsistencies between the results derived from different studies, there were some similarities regarding dietary patterns identified among children [22]. In the present meta-analysis, the consumption of fast food and snacks had a low prevalence. This finding was notable and justified only in primary schools.

A meta-analysis conducted by Babashahi et al. [23] investigated the pooled daily servings of the most common processed food consumed by children. Their meta-analysis consisted of 10 papers including 67,093 participants. The highest consumption was related to the sugars and sweets group followed by oils and then biscuits and cakes [23]. However, the present meta-analysis investigated unhealthy diet patterns and also in students who might not necessarily be children.

The limitations of the study were high heterogeneity and the possibility of publication bias. In spite of existing these limitations, subgroup analysis showed a lower heterogeneity. However, this study presented pooled prevalence rates for the first time in Iran for each type of unhealthy diet among the students.

## 5. Conclusions

The present systematic review found “breakfast skipper” as the most commonly investigated pattern of unhealthy diet, and as well, the meta-analysis part reported a pooled prevalence of 28% for unhealthy diet among the Iranian students. According to the different types of unhealthy diet patterns, the pooled prevalence rate varied from 6% to 75%. Although there was uncertainty regarding the pooled evidence, the whole of the mentioned range was clinically important. Therefore, planning for the reduction of such behaviors is strongly recommended in the present target population. Decisions and plans should be on the basis of the patterns.

## Figures and Tables

**Table 1:** Baseline characteristics of the eligible studies (six studies with nine unhealthy diet groups).

Study	Year	University student	Sample size	Event	Age^1^	Type of unhealthy diet	Male proportion	Quality score^2^
Azadbakht L.	2011	Yes	289	150	21	Breakfast skipper	0	6
Azadbakht L.	2011	Yes	289	23	21	Fast food	0	
Azadbakht L.	2011	Yes	289	217	21	Hydrogenated oil consumption	0	
Azadbakht L.	2011	Yes	289	46	21	Salty snacks	0	
Azadbakht L.	2011	Yes	289	104	21	Sweetened beverages	0	
Amini M.	2014	No	761	145	6.4	Breakfast skipper	0.50	7
Amini M.	2014	No	761	141	6.4	Salty snacks	0.50	
Amini M.	2014	No	761	365	6.4	Sweetened beverages	0.50	
Asadi M.	2014	No	510	75	15	Poor nutrition (always)	0.51	6
Safiri S.	2016	No	13486	4269	12.47	Breakfast skipper	0.51	6
Safiri S.	2016	No	13486	1450	12.47	Dinner skipper	0.51	
Safiri S.	2016	No	13486	382	12.47	Fast food	0.51	
Safiri S.	2016	No	13486	1170	12.47	Lunch skipper	0.51	
Safiri S.	2016	No	13486	1719	12.47	Salty snacks	0.51	
Safiri S.	2016	No	13486	3433	12.47	Sweetened beverages	0.51	
Safiri S.	2016	No	13486	4541	12.47	Sweets	0.51	
Shahsanai A.	2018	No	446	281	13.4	Breakfast skipper	1	6
Shahsanai A.	2018	No	446	240	13.4	Dinner skipper	1	
Shahsanai A.	2018	No	446	34	13.4	Fast food	1	
Shahsanai A.	2018	No	446	194	13.4	Lunch skipper	1	
Shahsanai A.	2018	No	446	61	13.4	Salty snacks	1	
Shahsanai A.	2018	No	446	72	13.4	Sweetened beverages	1	
Soheilipour F.	2018	No	829	256	9.77	Breakfast skipper	0.45	5

(1) Mean or midpoint of the reported range. (2) 5-6 scores: satisfactory, 7-8 scores: good, considering that the comparability score based on adjusting confounders (two points) was not applicable, and instead, we added one point for the studies had subgroups for types of unhealthy diet.

## Data Availability

The data that support the findings of this study are available from the corresponding author upon reasonable request.
